# Assessment of Quality of Life and Stress Levels Among Arab Mothers of Children With Congenital Heart Disease in the United Arab Emirates

**DOI:** 10.7759/cureus.85222

**Published:** 2025-06-02

**Authors:** Aya Abdullah Bin Taleb, Manar M Darwish, Charu Sharma, Sania Al Hamad, Ekhlass Mohammed Mohammed, Fadima Elmi, Venkatachalam Karuppaswamy, Mohammed AlSamri, Emad Masuadi, Elhadi H Aburawi

**Affiliations:** 1 Department of Pediatrics, Tawam Hospital, Al Ain, ARE; 2 Department of Internal Medicine, College of Medicine and Health Sciences, United Arab Emirates University, Al Ain, ARE; 3 Department of Pediatrics, College of Medicine and Health Sciences, United Arab Emirates University, Al Ain, ARE; 4 Department of Internal Medicine, Lincoln Medical Center, New York, USA; 5 Department of Public Health Institute, College of Medicine and Health Sciences, United Arab Emirates University, Al Ain, ARE

**Keywords:** children, chronic illness, health-related quality of life, mother stress, parents

## Abstract

Objectives: The quality of life (QOL) of mothers of children with chronic illnesses has become increasingly important, particularly with improved survival rates and reduced mortality. This study evaluated the stress levels and health-related QOL in families of children with congenital heart disease (CHD).

Methods: This cross-sectional study was conducted at a primary hospital in the United Arab Emirates specializing in children's heart diseases. A total of 205 Arabic mothers of children with heart disease were recruited. Data on QOL and disease-specific factors were collected through standardized assessments and validated measures in Arabic.

Results: According to the PSS, 178 (86%) of participants reported moderate stress, while 14 (6.8%) reported high stress. Substantial effects were observed in mothers' physical health, psychological well-being, environmental factors, and social relationships, with the strongest associations between the social and environmental domains. No significant differences in QOL were found between mothers of children with cyanotic and non-cyanotic CHD.

Conclusion: The study concluded that mothers of children with CHD generally experience moderate stress. No significant differences in stress levels were observed between mothers of children with cyanotic and non-cyanotic CHD. These results highlight the need to address stress and improve QOL for mothers of children with CHD.

## Introduction

Congenital heart diseases (CHDs) are structural heart conditions present at birth that affect the heart anatomy. Approximately 90% of children with CHD have the potential to reach adulthood and require ongoing specialized care throughout their lives [1]. This care must address various aspects, including physical activity, nutrition, and neurodevelopmental needs. Furthermore, individuals with CHD may face cognitive, social, and behavioral challenges that can affect their daily quality of life (QOL) and long-term functioning.

Long-term health conditions can substantially increase stress in both children and their caregivers. Studies have demonstrated that children with chronic conditions are twice as likely to develop mental health disorders as their healthy counterparts, with this risk increasing threefold in children with disabilities compared to those without additional health complications [2]. Studies have demonstrated that chronic illnesses adversely affect the health-related quality of life (HRQOL) of parents caring for children with disabilities. Parents of children with disabilities often experience increased stress, anxiety, and emotional strain due to constant caregiving demands. This ongoing responsibility can lead to a reduction in their physical health as they may neglect their own well-being while focusing on the needs of their children. Furthermore, the psychological toll of managing the complex healthcare needs, educational challenges, and social difficulties that arise when raising children with disabilities can result in feelings of isolation, frustration, and a decreased sense of personal accomplishment. Parents of children with chronic conditions frequently encounter a range of challenges across physical, social, emotional, and financial domains. Consequently, it is crucial to assess the psychological, psychosocial, and physical well-being of parents, as caregiving for children with chronic illnesses requires substantial family adjustment and ongoing parental support. Recognizing and addressing the needs of the family have been shown to improve medical outcomes for both parents and children. Of particular importance is the role of mothers, whose functioning and well-being are integral to the family’s adaptation to chronic pediatric conditions [3].

## Materials and methods

Study participants

This cross-sectional study was conducted at Tawam Hospital in the United Arab Emirates (UAE) over a 24-month period, from 2021 to 2023. A total of 205 mothers of children with CHD who met the inclusion criteria were recruited. The participants were recruited consecutively from the list that we obtained from the medical records of the hospital. The interviews were conducted by trained medical professionals in the outpatient clinics. The inclusion criteria were as follows: the child had been diagnosed with CHD by a pediatric cardiologist; the child’s age ranged from one month to 15 years; the mother was Arabic-speaking and provided both written and verbal informed consent to participate in the study; and the mother and child resided in the UAE. The exclusion criteria were as follows: mothers of children with CHD who also had other severe chronic illnesses that could potentially interfere with their ability to participate in the study or affect their health outcomes, and mothers diagnosed with psychiatric disorders. Clinical data, including the type of CHD and management strategies, were collected from attending cardiologists and medical records.

Data collection

Personal interviews were conducted with all the participating mothers to ensure that they met the inclusion criteria, fully understood the questionnaire items, and provided reliable and accurate responses. Subsequently, mothers self-reported their HRQOL.

The study used the World Health Organization Quality of Life-BREF (WHOQOL-BREF) assessment [4]. The WHOQOL-BREF is an abbreviated version of the comprehensive WHOQOL-100 instrument that consists of 26 items designed for the quick evaluation of health-related functions across four domains. It has demonstrated reliability and validity across various cultural groups, including the general Arab population, confirming its cross-cultural applicability. The Arabic version of the WHOQOL-BREF was used in this study [5]. Participants’ QOL was evaluated in terms of physical health, psychological well-being, social relationships, and environmental factors.

The WHOQOL-BREF uses a five-point scale, where one indicates "strongly agree" and five represents "strongly disagree." The mean score of each subscale contributed to the overall HRQOL score. Raw scores from the four domains were transformed from a four to 20 scale to a zero to 100 scale, with 100 indicating the highest QOL and zero representing the lowest. Higher scores reflect better QOL. Additional questions were included to gather sociodemographic data from the mothers, including age, gender, educational level, marital status, income, and disease-related information.

The Perceived Stress Scale (PSS) assessed participants' stress perception. This 14-item survey evaluates stressful experiences, with higher scores indicating greater stress. Each item is scored from zero to four, yielding a total from zero to 56, categorized as low stress: zero to 18, moderate stress: 19 to 37, and high stress: 38 to 56.

Statistical analysis

Data were entered and analyzed using IBM SPSS Statistics for Windows, Version 29 (Released 2023; IBM Corp., Armonk, New York, United States). Categorical variables are presented as frequencies and percentages, providing a clear overview of the distribution of different categories within the dataset. Numerical variables are summarized using means and standard deviations, offering insights into the central tendency and variability of the data. Analysis of variance (ANOVA) was used to compare the QOL scores and their domains across different stress levels. An unpaired t-test was used to compare perceived stress and QOL domains between mothers of children with cyanotic and non-cyanotic CHD. Statistical significance was set at a p-value of less than 0.05.

## Results

Demographic and socio-economic characteristics of the participants

The study included 205 mothers, with a mean age of 30.2 ± 6.2 years at the time of their child's birth. Table 1 summarizes the demographic and socioeconomic characteristics of the participating mothers. Most fathers (n = 157, 76.6%) and mothers (n = 167, 81.5%) were Emirati. The largest age group for fathers (n = 68, 34.2%) was 35 years or older, and for mothers (n = 68, 33.2%) was 26 to 30 years at the time of the child's birth. The most common educational attainment for both fathers (n = 98, 48.5%) and mothers (n = 95, 46.6%) was a university degree or higher. Regarding family income, 115 mothers (56.7%) reported a monthly income between 10,000 AED and 30,000 AED. Most mothers (n = 200, 98.0%) were married, with a small proportion divorced, widowed, or separated. A very small percentage of mothers (n = 4, 2.0%) reported having a psychiatric condition.

**Table 1:** Demographic and socio-economic characteristics of the participants

Characteristics		N	%
Father's age when the child is born (Years)	≤25	21	10.6
	26 – 30	55	27.6
	31 – 35	55	27.6
	35	68	34.2
Mother's age when the child is born (Years)	≤25	51	24.9
	26 – 30	68	33.2
	31 – 35	43	21.0
	35	43	21.0
Father's Nationality	Emirati	157	76.6
	Non-Emirati	48	23.4
Mother's Nationality	Emirati	167	81.5
	Non-Emirati	38	18.5
Father's education	Below secondary	27	13.4
	Secondary certificate	77	38.1
	University degree‎/postgrad	98	48.5
Mother's education	Below secondary	20	9.8
	Secondary certificate	89	43.6
	University degree‎/postgrad	95	46.6
Family income (N=mothers)	<10K AED	26	12.8
	10K – 30K AED	115	56.7
	30K – 50K AED	53	26.1
	>50K AED	9	4.4
Mother's marital status	Married	200	98.0
	Divorced	2	1.0
	Widowed	1	0.5
	Separated	1	0.5
Mothers with psychiatric disease	Yes	4	2.0
	No	195	98.0

Clinical characteristics of children with congenital heart disease

Further analysis was conducted among the mothers of the cyanotic and non-cyanotic groups to determine if the blue coloration of their children's lips had any impact on their stress and QoL. The results showed no significant difference in the stress levels or QoL between mothers' perceptions of the cyanotic and non-cyanotic groups, despite the presence of blue coloration in the children's lips.

Table 2 presents the clinical characteristics of children with CHD who participated in this study. The majority of children (n = 151, 74.0%) were diagnosed with non-cyanotic CHD, and 107 (52.2%) were male. The mean age of the children was 41.1 ± 38.6 months, with 48 (24.0%) having been born preterm. Additionally, 89 (43.6%) of the children had a history of cardiac surgery, and 34 (17.0%) had a history of other comorbidities.

**Table 2:** Characteristics of participating children with congenital heart disease (CHD)

Variables	Categories	N	%
Patient diagnosis	Non-cyanotic CHD	151	74.0
	Cyanotic	53	26.0
Age (months)	0 - < 12	38	18.5
	12 - < 24	41	20.0
	24 - < 60	80	39.0
	60+	46	22.4
Gender	Male	107	52.2
	Female	98	47.8
Preterm	Yes	48	24.0
	No	152	76.0
History of previous cardiac surgery	Yes	89	43.6
	No	115	56.4
History of other comorbidities	Yes	34	17.0
	No	166	83.0

Table 3 presents the prevalence of stress among mothers of children with CHD as measured using the Perceived Stress Scale. The scale ranged from zero to 49, with a mean (SD) of 29.7 (6.4). Additionally, the table provides the prevalence of stress categorized into three levels: low, moderate, and high. Overall, the majority of mothers experienced moderate stress (178, 86.8%), followed by high stress (14, 6.8%) and low stress (13, 6.4%).

**Table 3:** The prevalence of stress in mothers of children with congenital heart disease (CHD)

Findings	Min	Max	Mean	SD
Perceived Stress Scale (Max 56)	0	49	29.7	6.4
			95% CI for the prevalence	
Perceived Stress Scale	N	Prevalence	Lower	Upper
Low stress	13	6.4%	3.6%	10.3%
Moderate stress	178	86.8%	81.7%	90.9%
High stress	14	6.8%	4.0%	10.9%
Total	205	100.0%		

Table 4 presents the association between the QOL domains and perceived stress levels among mothers of children with CHD. Overall, there was a trend toward decreasing QOL scores with increasing levels of perceived stress across most domains, although the differences were not statistically significant for all domains. The P-values suggest that the association between perceived stress and QOL is not significant for some domains (e.g., social relationships, environment, and general well-being), while it approaches significance for others (e.g., physical health and psychological well-being; p-value = 0.081 and 0.087, respectively).

**Table 4:** The association between quality of life and perceived stress The p-value was considered significant at p<0.05, 0.001. QOL: quality of life

QOL domain	Perceived stress	N	Mean	SD	P-value
Physical health	Low stress	13	67.3	11.7	0.081
	Moderate stress	177	61.4	12.0	
	High stress	14	56.6	16.7	
	Total	204	61.4	12.4	
Psychological	Low stress	13	61.5	5.7	0.087
	Moderate stress	177	57.6	8.3	
	High stress	14	54.5	9.9	
	Total	204	57.6	8.3	
Social relationships	Low stress	13	75.0	7.6	0.992
	Moderate stress	178	75.6	16.1	
	High stress	14	75.6	22.8	
	Total	205	75.6	16.2	
Environment	Low stress	13	73.1	13.2	0.833
	Moderate stress	178	75.0	12.4	
	High stress	14	73.9	13.9	
	Total	205	74.8	12.5	
In general	Low stress	13	76.9	17.6	0.762
	Moderate stress	178	74.9	15.4	
	High stress	14	72.3	26.0	
	Total	205	74.9	16.3	

Quality of life among mothers of children with cyanotic congenital heart disease compared to mothers of children with non-cyanotic congenital heart disease

Table 5 compares the perceived stress scale and QOL domains between mothers of children with non-cyanotic and cyanotic CHD. Overall, the table suggests that there were no statistically significant differences between mothers of children with non-cyanotic and cyanotic CHD in terms of perceived stress scale and QOL domains. However, mothers of children with non-cyanotic CHD tended to have higher QOL scores for psychological well-being and social relationships (p = 0.065 and p = 0.091, respectively).

**Table 5:** Comparison of perceived stress scale and QOL domains between mothers of children with cyanotic and non-cyanotic CHD The p-value was considered significant at p<0.05, 0.001. QOL: quality of life; CHD: congenital heart disease

Domains	Non-cyanotic (n = 151)	Cyanotic (n = 53)	P-value
	mean ± SD	mean ± SD	
Perceived Stress Scale	29.5 ± 6.9	30.2± 4.9	0.524
Physical health	62.1 ± 13.5	59.8 ± 8.5	0.159
Psychological	58.4 ± 8.6	56 ± 6.7	0.065
Social relationships	76.7 ± 16	72.3 ± 16.6	0.091
Environment	75.7 ± 12.7	72.6 ± 12	0.126
In general	74.8 ± 16.7	75.2 ±15.4	0.854

## Discussion

Congenital heart disease is a debilitating chronic condition that affects the pediatric population [6]. Parents, particularly those serving as primary caregivers, face considerable challenges in maintaining a stable professional life due to the additional physical, emotional, and financial responsibilities associated with caring for a child with a chronic illness. Studies have demonstrated that parents of children with chronic illnesses often develop stronger social bonds within the family, which serve as a crucial support system and help them develop coping skills to manage their child's medical condition [7,8]. Parents of children with heart disease, in particular, report experiencing higher levels of stress than parents of children without health conditions [9,10].

In this cross-sectional study, the WHOQOL-BREF was used to assess the QOL of participants across four key domains: physical, social, psychological, and environmental well-being. This tool provides a comprehensive evaluation of individuals' experiences and perceptions in these areas, allowing for a nuanced understanding of their overall QOL. The results revealed that mothers of children with cyanotic CHD scored lower on these measures than mothers of children with non-cyanotic CHD. These findings are consistent with those of previous studies that have shown that parents of children with CHD report substantially lower QOL scores than parents of children with other chronic conditions or less severe illnesses [11,12].

One study revealed that 25% of mothers of children with CHD experienced persistently low mental HRQOL throughout their children’s development [13]. However, the focus of this study was not limited to mental health but encompassed all domains of QOL. Our findings indicate that while all aspects of QOL for mothers of children with CHD were notably affected, the social and environmental domains were most substantially impacted, with mean scores of 75.6 ± 16.2 and 74.8 ± 12.5, respectively. Social relationships and personal connections, as well as the social support systems available to these mothers, were particularly influenced. In the environmental domain, financial strain and stress within the home environment were the major contributing factors. These results contradict those of previous studies, suggesting that maternal stress primarily affects physical health and psychological well-being [14]. This underscores the need for targeted interventions to enhance social support systems and to create a more positive and supportive environment for mothers of children with chronic conditions.

Another study reported that parents of newborns with cyanotic CHD experience significantly high levels of stress, primarily driven by the emotional burden, uncertainty about the child’s health, and the challenges of managing medical treatments. These parents also report elevated levels of negative emotions such as anxiety, depression, and helplessness. Family and community support emerged as one of the most critical resources for parents, as it helped mitigate some of the emotional strain. Parents who had access to strong social networks experienced lower levels of stress and were better able to manage their emotions [15].

A study examined the differences between parental and self-reported assessments of QoL in Arab children and explored the factors affecting the QoL of children with CHD. The study reported that children with CHD exhibit reduced QoL, particularly in domains related to physical functioning and emotional health, and the role of family support for better emotional outcomes for these children [16].

The perception of QoL in children and adolescents with CHD based on self-reports and proxy reports from parents. The study emphasizes the need for comprehensive care for children and adolescents with congenital heart disease, encompassing not only medical treatment but also psychosocial interventions. Improving emotional health and social integration and providing family-centered care could help mitigate the negative impact of CHD on their quality of life [17].

In this study, we used the WHOQOL-BREF and PSS, which are widely accepted, culturally adapted, and validated tools. We included 205 Arabic-speaking mothers of children with CHD, which is quite a reasonable number, making it a valuable contribution. These findings have huge clinical implications, as they can inform healthcare providers about the need for targeted psychosocial interventions for caregivers. In the UAE, there is still a stigma associated with children born with CHD, which can be exacerbated if the CHD is visible or involves frequent hospitalizations, leading to social isolation and judgment. The CHD may be viewed as a rare and unfortunate condition, leading to heightened anxiety and stress for mothers. Cultural beliefs around perfection and ideal family structures can make mothers feel as though their child’s illness reflects poorly on them or their family. Extended families often play a major role in the UAE’s culture, and support from family members may be crucial in reducing stress. However, more modern and educated views may emphasize the medical advancements and treatment options available, potentially reducing maternal anxiety and stress.

In the UAE, mothers of children with CHD have access to various support systems, including advanced healthcare services, religious and cultural support, and community resources. Furthermore, availability of a home care nurse and rehab center appointments is available. Mental health services, educational programs, and online communities also provide critical information and peer support. Family and extended family often play a significant role in caregiving. These are all the keys to further reducing maternal stress and improving quality of life for mothers.

Interestingly, our study found no significant differences in stress levels between mothers of children with cyanotic and non-cyanotic CHD. However, the average stress scores of mothers of children with non-cyanotic CHD were higher than those of mothers of children with cyanotic CHD. Several factors may have contributed to this finding, such as the mother's understanding of the specific type of CHD and the inherent distinctions between cyanotic and non-cyanotic forms. Additionally, cultural and religious factors may influence the level of awareness regarding CHD and its associated complications, which could impact the stress experienced by the mothers.

Limitations

This study has some limitations that must be acknowledged. A primary limitation was the reliance on assessments provided solely by mothers, excluding potential insights from fathers, which may also influence stress levels. Additionally, the study did not examine the impact of factors such as the age of children with CHD or the duration of the illness on the family’s perception of QOL. Another limitation is the relatively small sample size, which was limited by challenges in recruiting participants to complete the questionnaires. Therefore, the findings should be considered preliminary. Further research with a larger sample size and long-term follow-up is necessary to gain a more comprehensive understanding of the impact of CHD on families.

## Conclusions

Based on the short-term results of this study, it can be concluded that mothers of children with CHD generally report moderate stress levels across all aspects of QOL. Furthermore, no significant differences in stress levels were found between mothers of children with cyanotic and non-cyanotic CHD. These findings underscore the need for future initiatives aimed at increasing the awareness and knowledge of CHD, along with providing targeted support, particularly in the social and environmental domains.
